# Impact of Opioid Analgesia and Inhalation Sedation Kalinox on Pain and Radial Artery Spasm during Transradial Coronary Angiography

**DOI:** 10.3390/jcm9092747

**Published:** 2020-08-25

**Authors:** Caroline Birgy, Antonin Trimaille, Nathan Messas, Jessica Ristorto, Anas Kayali, Benjamin Marchandot, Thomas Cardi, Sébastien Hess, Marion Kibler, Laurence Jesel, Patrick Ohlmann, Olivier Morel

**Affiliations:** 1 Pôle d’Activité Médico-Chirurgicale Cardiovasculaire, Hôpitaux Universitaires de Strasbourg, Université de Strasbourg, 67000 Strasbourg, France; c.birgy@live.fr (C.B.); antonin.trimaille@chru-strasbourg.fr (A.T.); haimmessas@hotmail.fr (N.M.); ristorto-jess@hotmail.fr (J.R.); anas.kayali@chru-strasbourg.fr (A.K.); benjamin.marchandot@gmail.com (B.M.); thomas.cardi@chru-strasbourg.fr (T.C.); sebastien.hess@chru-strasbourg.fr (S.H.); marion.kibler@chru-strasbourg.fr (M.K.); laurence.jesel@chru-strasbourg.fr (L.J.); patrick.ohlmann@chru-strasbourg.fr (P.O.); 2 INSERM UMR-1260 Regenerative Nanomedicine, Université de Strasbourg, 67000 Strasbourg, France

**Keywords:** percutaneous coronary intervention, radial artery spasm, analgesia, MEOPA, Kalinox, Entonox, N_2_O/O_2_, nitrous oxide sedation

## Abstract

With respect to the transfemoral approach, transradial procedures enable a drastic reduction of bleeding events and are associated with a reduction of mortality. Radial artery spasm (RAS) is one of the most common complications and may lead to patient discomfort and procedural failure. Currently, there is no consensus on the optimal sedation protocol to avoid RAS. The aim of this study was to investigate the respective impact of opioids analgesia and inhalation sedation with a 50% nitrous oxide/oxygen premix (Kalinox) on pain and occurrence of RAS during transradial coronary procedures. Consecutive patients undergoing transradial coronary angiography were prospectively enrolled in one, single center observational study (Nouvel Hôpital Civil, Strasbourg, France). Patients received opioids analgesia or inhalation sedation with Kalinox. The primary endpoints of the study were the incidence of a pain scale ≥5/10 and the occurrence of RAS. The secondary endpoints were the incidence of side effects. A total of 325 patients were enrolled (185 in the opioids analgesia group, 140 in the Kalinox group). RAS and pain scale ≥5 rates were not significantly different in the opioids analgesia and Kalinox groups (respectively 13.5% vs. 10.0% and 16.2% vs. 11.4%). Headache was more frequently observed in the Kalinox group (6.4% vs. 0.0%; *p* = 0.002). By multivariate analysis, female gender, BMI <25 kg/m^2^, puncture difficulty, the use of plastic needle and 6F sheath were identified as independent predictors of RAS. Procedural inhalation sedation by Kalinox is as safe as opioids analgesia during transradial coronary angiography.

## 1. Introduction

With respect to the transfemoral approach, transradial procedures enable a drastic reduction of bleeding events and are associated with a reduction of mortality [1,2]. Radial artery spasm (RAS) is one of the most common complications and may lead to patient discomfort and procedural failure. Currently, there is no consensus on the optimal sedation protocol to avoid RAS [3,4]. The aim of sedation and analgesia in medical procedures like percutaneous coronary intervention (PCI) is to reduce the pain and anxiety caused by the procedure with the use of medications with sedatives, analgesics and amnetics effects.

Opioids are commonly used during PCI but they have numerous side effects including the depression of the respiratory system. Owing to their narrow therapeutic window, restriction of their use has been advocated by regulatory agencies and their administration is restricted to anesthesiologists or skilled, critical care physicians. Another important concern that could preclude the systematic use of opioids analgesia during PCI is the recent description of a limited absorption of P2Y_12_ inhibitors by opioids as a possible reduction of gut motility [5].

Kalinox is an odorless gas used for procedural sedation and analgesia, typically administered as a 50-50% nitrous oxide-oxygen mixture (N_2_O/O_2_) to avoid hypoxemia. It is an effective analgesic/anxiolytic agent causing central nervous system depression and euphoria with little effect on the respiratory system. There are many attractive qualities of Kalinox due to its low solubility in the blood which allows a rapid onset of action in the brain as well as rapid clearance through the lungs shortly after administration. It provides a minor amnestic effect [6]. Kalinox has demonstrated its analgesic efficacy and safety in various procedures associated with mild to moderate pain including minor surgery, emergency transportation, venous cannulation, colonoscopy, dentistry, percutaneous liver biopsy or percutaneous renal biopsy [7,8,9]. Up to now, the use of Kalinox has not been investigated in coronary interventions with a rarity of data exploring its use during cardioversion [10].

The present study was therefore designed to investigate the feasibility and efficacy on pain and RAS of inhalation sedation by 50% nitrous oxide/oxygen premix (Kalinox) in comparison with opioid analgesia during transradial procedures. In addition, the secondary objective was to assess the predictors of RAS and pain after PCI.

## 2. Methods

### 2.1. Patients

From September 2016 to February 2017, patients undergoing elective or urgent transradial coronary procedures were prospectively enrolled in a high volume (1560 PCI procedures in 2016) PCI center (Nouvel Hôpital Civil, Strasbourg, France). Patients with contraindications (listed in Appendix A) for the use of Kalinox were excluded. Patients received the current analgesia protocol of our center, i.e., opioids analgesia (alfentanil 200 µg or morphine 2 mg intravenous), or inhalation sedation by 50% nitrous oxide/oxygen premix (Kalinox). Operators were encouraged to treat one of two patients with Kalinox and one of two with opioids analgesia. In case of Kalinox intolerance, administration of opioids was permitted. If required to complete the procedure, additional doses of opioids were allowed in patients who received opioid analgesia. All procedures were performed in rooms equipped with a gas scavenging system to limit the medical personnel exposure to nitrous oxide.

Patients’ baseline characteristics were recorded: age, gender, size, weight, history of hyperlipidemia, smoking habits, hypertension, diabetes mellitus, coronary arterial disease, peripheral arterial disease (PAD), chronic kidney disease (CKD), medication and materials used during PCI.

For all patients, a puncture was made with a metallic or plastic 19-gauge needle after local anesthesia with 1–4 mL lidocaine 1%. Numbers of puncture were recorded. A 7 cm, 4–6F (hydrophilic coated sheath Radifocus^®^ Introducer II set, Terumo, Tokio, Japan) was used and patients in both groups received 3000 to 6000 units heparin according to body weight, 1 mg of isosorbide dinitrate and 1 mg of nicardipine through the radial sheath immediately after insertion. If required, another administration of nicardipine was added. A 0.038 inch guidewire was used (Boston Scientific, Boston, MA, USA). The procedure time was calculated as the time from local anesthesia to the removal of the sheath. Postprocedural hemostasis was achieved by placing of a wrist clamp device (TR Band, Terumo, Tokio, Japan).

All participants gave their informed written consent for the recording of their data in an anonymized database before the procedure. The study was approved by the University of Strasbourg bioethics committee (reference number: CE-2020-49). The study protocol conforms to the ethical guidelines of the 1975 Declaration of Helsinki.

### 2.2. Clinical Endpoints

Patients’ perceived discomfort was assessed in the evening after the procedure or the day after with a visual analogue scale (VAS) whereby the patient graded the discomfort on a scale of 0 to 10 (0 corresponding to “no discomfort” and 10 to “extreme pain and discomfort”). RAS severity was categorized as none, moderate and severe. Spasm was identified by appearance of marked resistance to the movement of the catheter or inability to further advance catheters with or without accompanying forearm pain.

An RAS risk score was calculated for all patients as recently proposed by Giannopulos and coworkers [11]. It consists of five weighted risk factors for radial artery spasm: body mass index (BMI) less than 25 kg/m^2^ (1 point), height less than 170 cm (1 point), current smoking (2 points), hypertension (2 points) and PAD (3 points). A predefined score of 4 or more is related to a high risk of RAS [11].

The primary endpoints of the study were the incidence of a pain scale greater than or equal to 5 and the occurrence of RAS. The secondary endpoint included the occurrence of the most frequent side effects of Kalinox such as nausea, vomiting, dizziness or faintness and headache.

### 2.3. Statistical Analysis

Categorical variables were expressed as counts and percentages. Continuous variables were reported as the mean +/− standard deviation (SD) according to their distribution. Categorical variables were compared with Chi-square tests or Fisher’s exact tests. Continuous variables were compared with the use of parametric (ANOVA) or nonparametric Mann-Whitney test as appropriate. To determine predictors of radial spasm and pain, regression analysis was performed. Variables with *p* < 0.05 in univariate analysis were entered into a stepwise ascending multivariate analysis. A *p* value < 0.05 was considered significant. Since Kalinox was never studied in PCI, the sample size could not be calculated. Based on previous studies on RAS, we estimated that a sample size of 300 patients would be adequate. Calculations were performed using SPSS 17.0 for Windows (SPSS Inc., Chicago, IL, USA).

## 3. Results

### 3.1. Patients and Baseline Characteristics

A total of 325 patients were prospectively enrolled. Among these, 185 patients benefited from opioids analgesia by alfentanil/morphine (OA group) and 140 patients from inhalation sedation by Kalinox (K group). Baseline demographics, clinical, biological and angiographic characteristics of the two groups are described in Table 1 and Table 2. A higher rate of diabetic patients could be evidenced in the OA group (31.4% vs. 21.4%; *p* = 0.032). Conversely, patients in the K group were more frequently smokers (30% vs. 15.7%; *p* = 0.002) and had a higher rate of peripheral artery disease history (12.1% vs. 4.9% *p* = 0.017). Importantly, RAS risk score was equivalent between the two groups (OA 2.4 ± 1.6 vs. K 2.6 ± 1.7; *p* = 0.46). Other characteristics (clinical presentation, extent of coronary artery disease, sheath size, procedural time) were equally balanced.

### 3.2. Radial Artery Spasm

**Figure 1:**
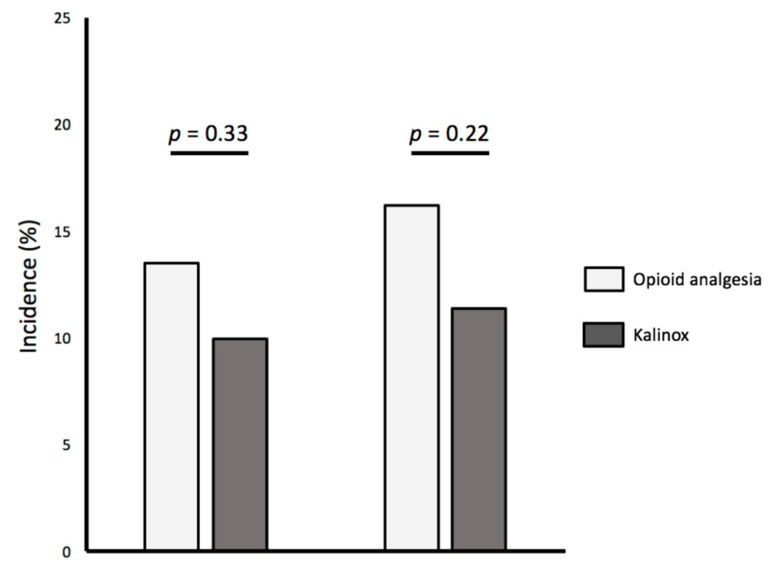
Incidence of Radial Spasm and Pain Scale ≥5 according to antalgic group.

The incidence of spasm was 12.0% (39/325). Similar rates of RAS were observed in the Kalinox group and in the opioid analgesia group (10.0% vs. 13.5%; *p* = 0.334) (Figure 1). By univariate analysis, female gender, lower body mass index, duration of procedure, puncture difficulty, 6F sheath, the use of plastic cathlon and pain scale ≥5 were associated with RAS. No impact of RAS score or high-risk RAS score on RAS could be evidenced. Conversely, nicardipine administration together with the use of 5F sheaths were associated with lower RAS (Table 3). By multivariate analysis, female gender, BMI < 25 kg/m^2^, puncture difficulty, the use of plastic needles and 6F sheaths were identified as independent predictors of RAS (Table 4).

### 3.3. Pain Scale

The incidence of pain scale ≥5 was 14.1% (46/325). As observed on RAS, similar rates of pain scale ≥5 were observed in the Kalinox group and in the opioid analgesia group (11.4% vs. 16.2%; *p* = 0.220) (Figure 1). By univariate analysis, female gender, angina and RAS were associated with a pain scale ≥5. No impact of RAS score or high-risk RAS score on pain scale ≥5 could be evidenced (Table 5). By multivariate analysis, angor as an indication of transradial coronary procedure and radial spasm were identified as independent predictors of a pain scale ≥5 (Table 6).

### 3.4. Safety and Side Effects

Overall, adverse effects were rare. No cases of respiratory depression requiring mechanical support were recorded. In one case, ventilation sedation by Kalinox had to be stopped due to intolerance. Re-administration of opioid to complete the procedure was noticed in 18.9% (35/185) in the OA group. Administration of Kalinox did not induce more nausea or vomiting (2.9% versus 1.6%; *p* = 0.44). Accordingly, comparable rates of dizziness or faintness could be observed between groups. Conversely, higher rate of headache could be evidenced in patients treated by Kalinox (Table 7).

## 4. Discussion

The main finding of this prospective study is the demonstration of the feasibility of ventilation sedation by Kalinox during transradial coronary procedures. With respect to our current protocol using opioid analgesia, we observed similar incidence of pain and RAS in patients undergoing the transradial approach under Kalinox. Not surprisingly, our findings confirm the deleterious impact of female gender, low body weight, puncture difficulty and large sheath on the occurrence of RAS.

### 4.1. Radial Artery Spams Incidence and Risk Factors

The transradial approach for left heart catheterization and PCI is increasingly used worldwide given its numerous advantages including lower bleeding rates, lower mortality, reduced mobilization time and shorter hospital stays [2]. Increased rates of access site crossover have been described with the radial approach and radial artery spasm has been identified as a major contributor of procedural failure or crossover. Moreover, radial artery spasm may lead to intimal wall injury, intimal thickening and radial occlusion hampering future procedures. The reported frequency of spasm in the transradial approach presents huge variations in the literature ranging from 4% to higher than 20% in line with the 12% reported in the present work [12,13]. Numerous factors predisposing to RAS have been identified in the published data including BMI < 25 kg/m^2^, height <170 cm, current smoking, PAD, age, complexity of the procedure, anatomical variations and large radial sheaths and catheter [11]. To allow a better identification of high risk patients, an RAS risk score has been recently proposed by Giannopoulos et al. based on five weighted variables (see methods) with a cut-off of four to predict spasm with a sensitivity of 84.5% and a specificity of 74.7% [11]. In the present cohort, we could only identify the noxious impact of BMI < 25 kg/m^2^ on RAS but other parameters (current smoker, arterial hypertension, height <170 cm, PAD) are nonrelevant. Not surprisingly, female gender was identified as an important risk factor of RAS. Important variations in the radial artery diameter between men and women have been described in the past (2.69 ± 0.40 mm in men and 2.43 ± 0.38 mm in women) [14] and radial artery-sheath mismatch (ratio between radial artery inner diameter and sheath outer diameter smaller than 1) has been evidenced as the strongest preprocedural predictors of RAS [12]. Of note the 6F radial sheath used in our study has an outer diameter of 2.62 mm which implies that a mismatch is present in at least 60% of the women [15].

Noxious connections between anxiety, pain, catecholamines release and RAS have been previously underlined. The possible impact of angina as a clinical presentation is more puzzling. Previous data have suggested that endothelial dysfunction, a key parameter during angina, and characterized by reduced production of nitric oxide (NO) and increased NO breakdown by reactive oxygen species may favor paradoxical vasoconstriction of the vessel and contribute to radial spasm. However, recent findings by van der Heiden failed to evidence any link between endothelial function as assessed by EndoPAT and RAS [12].

Along with a technical approach that enables the reduction of spasm such as the downsizing of sheaths and catheters or the use of hydrophilic coated sheaths, the use of appropriate sedation together with vasodilation appears to be an intuitive approach to limit spasm and patient discomfort. In line with this view, data by Deftereos and coworkers have emphasized that routine administration of relatively low doses of an opioid (fentanyl) in combination with benzodiazepine is associated with a significant reduction of spasm, site crossover and patient discomfort [4]. By contrast, another investigation did not evidence any difference in the occurrence of RAS between nitroglycerin alone and nitroglycerin plus midazolam when hydrophilic small sheaths were used [13].

### 4.2. Kalinox Effects

With respect to opioid analgesia, inhalation sedation by 50% nitrous oxide/oxygen premix (Kalinox) presents several advantages: (i) its administration does not require the presence of an anesthesiologist and can be conducted under the supervision of a trained nurse with conditions and tools of monitoring already used for PCI; (ii) contraindications are limited and allow widespread use (see Appendix A); (iii) its price is affordable. In our experience, procedures could be achieved in all but one patient under Kalinox whereas 18.9% of the patients in the opioid analgesia required additional administration of opioids. The Kalinox side effect profile was evaluated versus placebo in a previous study in children [16]. A similar side effect profile in both groups was found with only two patients under Kalinox who reported dizziness requiring treatment withdrawal.

The analgesic properties of nitrous oxide (N_2_O) have been described for more than 150 years and were first exploited in dental surgery, obstetrics and more recently pediatric settings [6]. Its impact on the cardiovascular system remains poorly investigated. Previous findings have suggested that 50/50 mixture of nitrous oxide does not cause significant changes in cardiac output and blood pressure. Although N_2_O has been reported to decrease myocardial contractility in vitro, it also simultaneously stimulates catecholamine release by the sympathetic nervous system hereby leaving cardiac output, blood pressure and heart rate relatively unchanged in vivo [6]. However, the ENIGMA trial (Evaluation of Nitrous Oxide in the Gas Mixture for Anesthesia) [17] sounds the alarm about a possible noxious impact of nitrous oxide on late cardiovascular events. Pathophysiological effects of nitrous oxide on the cardiovascular system may include the inhibition of methionine synthase with resulting hyperhomocysteinemia and endothelial dysfunction [18]. In this trial, the use of nitrous oxide increased the risk of myocardial infarction (OR 1.50, 95% CI 1.01–2.51; *p* = 0.04) at long-term follow-up but not at 30 days. However more recently, the ENIGMA-II, a powered study enrolling 7112 non-cardiac-surgery patients at risk of perioperative cardiovascular events, demonstrated the long-term safety of nitrous oxide administration without any elevation of death, myocardial infarction and stroke rates [18]. Altogether, these data support the long-term safety of nitrous oxide administration for the patient. However, operators should watch out for air embolism with a rigorous prevention of air presence in the nitrous oxide circuit. Moreover, the cath lab should be equipped with gas scavenging systems to limit the exposure of medical personnel to nitrous oxide. Chronic exposure to nitrous oxide could reduce fertility and increase the rate of spontaneous abortion in female workers [19].

Other anesthetics without needles have been studied such as lidocaine/prilocaine mixture presented as an ointment which have been shown as an alternative of lidocaine infiltration in transradial cardiac catheterization [20]. However, this anesthetic has never been compared with the 50% nitrous oxide/oxygen premix (Kalinox).

### 4.3. Study Limitations

The present work is monocentric and has the inherent limitations of any small series. The limited size of the cohort could have hampered the detection of RAS. The study was not randomized.

Spasm cannot always be defined with objectivity and different definitions have been provided in the past. Angiographic confirmation of the spasm was not performed. In addition, since the PCI operators knew the analgesic regimen of the patient, we cannot exclude a bias in line with the evaluation of RAS. However, angiographic spasm is a common finding (up to 75% of cases) and there is a weak relation between angiographic spasm and procedural outcomes [21]. The radial artery size was not systematically recorded in this study. Nevertheless, a small radial artery makes the radial artery catheterization difficult and could for this reason increase radial artery spasm. The risk factors included in a high RAS risk score—such as female sex, small patient size or BMI—are related with small radial artery size and could indirectly explain the increase of this complication.

An imbalance in diabetic or smoking status was evidenced between groups. Since current smoking is an important factor of RAS, we could not exclude that the higher rate of smoking in the Kalinox group may have blunted the protective effect of Kalinox on spasm.

Patients evaluated their pain several hours after the procedure which may lead to a reduction in the intensity of the pain actually felt, especially when taken into account that Kalinox has an amnestic effect. Anxiety score was not assessed. Other possible impacts of nitrous oxide on hyperemia such as those induced during fractional flow reserve measurement should be investigated in appropriate trials.

## 5. Conclusions

Procedural inhalation sedation by Kalinox is as safe as opioids analgesia during transradial coronary angiography. Patient-administered N_2_O/O_2_ inhalation provides a safe and effective analgesia at a reasonable cost with a low rate of side effects. Further studies are needed to definitively recommend Kalinox for routine use during transradial PCI.

## Figures and Tables

**Table 1:** Baseline demographic clinical and biological characteristics according to antalgic group.

Variable	Opioid Analgesia (*n* = 185)	Kalinox (*n* = 140)	*p* Value
Age—years	66.8 ± 11.9	63.2 ± 11.3	0.060
Size—cm	169.7 ± 9.2	171.4 ± 9.4	0.104
Weight—kg	80.4 ± 17.1	81.3 ± 15.5	0.618
Male—*n* (%)	128 (69.2)	110 (78.6)	0.059
Diabetes mellitus—*n* (%)	58 (31.4)	30 (21.4)	0.032
Insulinotherapy—*n* (%)	15 (8.2)	9 (6.4)	0.557
Hypertension—*n* (%)	105 (56.8)	75 (53.6)	0.567
Smoking—*n* (%)	93 (50.3)	88 (62.9)	0.024
Current smoking—*n* (%)	29 (15.7)	42 (30.0)	0.002
Hyperlipidemia—*n* (%)	95 (51.4)	68 (48.6)	0.620
Known CAD—*n* (%)	87 (47)	63 (45)	0.717
PAD—*n* (%)	9 (4.9)	17 (12.1)	0.017
RAS score—IU	2.4 ± 1.6	2.6 ± 1.7	0.455
LVEF—*n* (%)	55.7 ± 10.3	56.2 ± 10.8	0.672
≤35%	3 (1.7)	1 (0.8)	
36–40%	7 (4.0)	3 (2.3)	0.713
41–50%	17 (9.8)	12 (9.2)	
≥50%	146 (84.4)	115 (87.8)	
eGFR—mL/min	74.9 ± 17.4	76.4 ± 17.9	0.437
eGFR—*n* (%)			
<30 mL/min	0	0	
30 to 60 mL/min	30 (16.5)	17 (12.6)	0.626
60 to 90 mL/min	82 (45.1)	63 (46.7)	
eGFR >90 mL/min	70 (38.5)	55 (40.7)	

Data are presented as mean ± standard deviation in case of any other indication. CAD, coronary artery disease; eGFR, estimated glomerular filtration; IU, international units; LVEF, left ventricular ejection function; PAD, peripheral artery disease; RAS score: radial artery spasm scores.

**Table 2:** Characteristics of transradial procedure according to antalgic group.

Variable	Opioid Analgesia (*n* = 185)	Kalinox (*n* = 140)	*p* Value
Indication of PCI— *n* (%)			
Heart failure	9 (4.9)	11 (7.9)	0.266
Programmed angioplasty	20 (10.8)	11 (7.9)	0.369
Post-angioplasty control	6 (3.2)	8 (5.7)	0.277
Pre-operative exam	12 (6.5)	9 (6.4)	0.983
Silent ischemia	53 (28.6)	36 (25.7)	0.557
NSTEMI	19 (10.3)	14 (10)	0.936
STEMI	3 (1.7)	2 (1.4)	0.732
Stable Angina	38 (20.5)	30 (21.4)	0.845
Valvular heart disease	14 (7.6)	11 (7.9)	0.923
Dyspnea	6 (3.2)	2 (1.4)	0.293
Heart rhythm disorder	2 (1.1)	7 (5)	0.033
Height of radial puncture—*n* (%)			
4F	3 (1.6)	4 (2.9)	0.447
5F	104 (56.2)	88 (62.9)	0.228
6F	79 (42.7)	48 (34.3)	0.124
Iode volume—mL	76.7 ± 69.3	86.6 ± 58.4	0.176
Procedural time—min	23 ± 17.4	22.5 ± 17.8	0.798
Height of introducer—*n* (%)			
4F	6 (3.2)	8 (5.7)	0.277
5F	110 (59.5)	90 (64.3)	0.376
6F	69 (37.3)	42 (30)	0.170
Type of introducer—*n* (%)			
Short	176 (95.1)	136 (97.1)	0.487
Long hydrophilic	9 (4.9)	4 (2.9)	0.360
Difficulty of puncture—*n* (%)			
One puncture	162 (87.6)	124 (88.6)	
One to three punctures	17 (9.2)	12 (8.6)	0.960
More than three	6 (3.2)	4 (2.9)	
Kind of needle—*n* (%)			
19 Gauges	155 (83.8)	124 (88.6)	0.220
M Coat	1 (0.5)	1 (0.7)	0.843
Plastic cathlon	29 (15.7)	15 (10.7)	0.195
Severity of CAD—*n* (%)			
1-vessel disease	75 (29.1)	72 (28.1)	0.791
2-vessel disease	58 (22.6)	43 (16.8)	0.100
3-vessel disease	30 (11.7)	30 (11.7)	0.997

Data are presented as mean ± standard deviation in case of any other indication. CAD, coronary artery disease; NSTEMI, non-ST segment elevation myocardial infarction; F, French; PCI, percutaneous coronary intervention; STEMI, ST segment elevation myocardial infarction.

**Table 3:** Univariate analysis for prediction of Radial Artery Spasm.

Variables	HR	CI 95%	*p* Value
Age < 50 years	1.21	0.39–3.69	0.734
BMI < 25 kg/m^2^	2.09	1.06–4.10	0.031
Height < 170 cm	1.26	0.65–2.45	0.492
Female gender	3.20	1.62–6.30	0.001
Diabetes mellitus	1.18	0.57–2.46	0.640
Insulinotherapy	0.96	0.27–3.39	0.960
Hypertension	0.97	0.50–1.89	0.940
Smoking	0.97	0.50–1.89	0.940
Current smoking	1.42	0.67–3.02	>0.350
Hyperlipidemia	1.10	0.57–2.14	0.760
Known CAD	1.87	0.95–3.68	0.060
PAD	1.27	0.41–3.88	0.670
LVEF	1.01	0.98–1.04	0.480
CKD	0.99	0.97–1.01	0.330
RAS score	1.13	0.94–1.36	0.192
High risk RAS score	1.40	0.69–2.84	0.339
Procedure duration	1.018	1.002–1.035	0.020
Kalinox use	0.71	0.355–1.424	0.336
Height of introducer			
5F	0.46	0.24–0.91	0.020
6F	2.62	1.33–5.16	0.005
Puncture difficulty	2.55	1.44–4.5	0.001
Supplementary dose of Nicardipine	0.4	0.21–0.75	0.005
Metallic 19 G Needle	0.31	0.14–0.67	0.003
Plastic cathlon	2.47	1.11–5.5	0.020
Pain Scale ≥5	3.28	1.46–7.38	0.004

CAD, coronary artery disease; CI, confidence interval; CKD, chronic kidney disease; HR, hazard ratio; LVEF, left ventricular ejection function; PAD, peripheral artery disease.

**Table 4:** Multivariate analysis for identification of Predictors of Radial Artery Spasm.

Variables	HR	CI 95%	*p* Value
Female gender	3.15	1.45–6.85	0.004
BMI < 25 kg/m^2^	2.09	0.999–4.40	0.050
Plastic cathlon	2.63	1.07–6.46	0.035
6F introducer	2.89	1.23–6.82	0.015
Puncture difficulty	2.14	1.13–4.04	0.019
Pain Scale ≥5	2.17	0.93–5.07	0.070
Procedure duration	1.01	0.99–1.03	0.300

BMI: body mass index; CI, confidence interval; HR: hazard ratio.

**Table 5:** Univariate analysis for prediction of Pain Scale ≥5.

Variables	HR	CI 95%	*p* Value
Age < 50 years	1.36	0.49–3.74	0.550
Weight < 60 kg	1.36	0.49–3.78	0.550
Female gender	2.15	1.12–4.10	0.020
Diabetes mellitus	1.07	0.53–2.15	0.830
Insulinotherapy	1.17	0.38–3.57	0.780
Hypertension	0.69	0.37–1.30	0.250
Smoking	0.94	0.50–1.77	0.860
Current smoking	1.15	0.55–2.40	0.700
Hyperlipidemia	1.48	0.78–2.79	0.220
Known CAD	0.87	0.46–1.64	0.670
PAD	0.74	0.21–2.57	0.640
LVEF	1.06	0.97–1.03	0.690
CKD	1.01	0.99–1.03	0.210
Angor	2.33	1.18–4.60	0.010
RAS score	1.10	0.92–1.32	0.260
High risk RAS score	1.40	0.69–2.84	0.339
Kalinox use	0.66	0.34–1.27	0.222
Procedure duration	1.01	0.99–1.02	0.230
Introducer 5F	0.52	0.28–0.98	0.040
Spasm	2.72	1.24–5.92	0.012

CAD, coronary artery disease; CI, confidence interval; CKD, chronic kidney disease; HR, hazard ratio; LVEF, left ventricular ejection function; PAD, peripheral artery disease.

**Table 6:** Multivariate analysis for prediction of Pain Scale ≥5.

Variables	HR	CI 95%	*p* Value
Female gender	1.81	0.92–3.55	0.083
Angor as indication of PCI	2.22	1.11–4.44	0.023
Radial Spasm	2.28	1.01–5.15	0.046

CI, confidence interval; HR, hazard ratio; PCI, percutaneous coronary intervention.

**Table 7:** Incidence of adverse effects according to antalgic group.

Variables	Opioid Analgesia (*n* = 185)	Kalinox (*n* = 140)	*p* Value
Nausea/vomiting—*n* (%)	3 (1.6)	4 (2.9)	0.44
Dizziness/faintness—*n* (%)	16 (8.6)	7 (5)	0.20
Headache—*n* (%)	0 (0.0)	9 (6.4)	0.002

## Appendix A. Contraindications for Use of Kalinox

▪ Hypersensitivity to the active substance.
▪ In patients with signs or symptoms of pneumothorax, pneumopericardium, severe emphysema, gas emboli or head injury.
▪ Maxillofacial injuries.
▪ Following deep sea diving with risk of decompression sickness (bubbles of nitrogen).
▪ Following treatment with heart lung machine or coronary bypass without heart lung machine.
▪ In patients recently having undergone intraocular injection of gas until the gas in question is fully absorbed, or within 3 months after the last injection of an intraocular gas, because the gas volume may increase in pressure/volume and consequently result in blindness.
▪ Patients with a severely dilated gastrointestinal tract.
▪ Following air encephalography.
▪ During middle ear, inner ear and sinus surgery.
▪ If air has been injected into the epidural space to determine the placement of the needle for epidural anesthesia.
▪ In patients showing signs of confusion or in some other way showing signs of increased intracranial pressure.
▪ Patients with untreated vitamin B12- or folic acid deficiency or diagnosed genetic disorder of the enzyme system involved in metabolism of these vitamins.
▪ Patients with a decreased level of consciousness or impaired ability to cooperate and follow instructions due to the risk that further sedation from the nitrous oxide may affect natural protective reflexes.
▪ Patients with facial injury where use of a face mask may present difficulties or risks.
